# Analysis of respiratory‐induced motion trajectories of individual liver segments in patients with hepatocellular carcinoma

**DOI:** 10.1002/acm2.14257

**Published:** 2024-02-01

**Authors:** Takahiro Kato, Kimihiro Takemasa, Tomohiro Ikeda, Hisanori Sakagami, Ryohei Kato, Yuki Narita, Sho Oyama, Shinya Komori, Hisashi Yamaguchi, Masao Murakami

**Affiliations:** ^1^ Department of Radiation Physics and Technology Southern Tohoku Proton Therapy Center Fukushima Japan; ^2^ Department of Radiological Sciences School of Health Sciences Fukushima Medical University Fukushima Japan; ^3^ Department of Radiation Oncology Southern Tohoku Proton Therapy Center Fukushima Japan; ^4^ Department of Minimally Invasive Surgical and Medical Oncology Fukushima Medical University Fukushima Japan

**Keywords:** hysteresis, liver segment, proton therapy, respiratory motion

## Abstract

**Purpose:**

To analyze the respiratory‐induced motion trajectories of each liver segment for hepatocellular carcinoma (HCC) to derive a more accurate internal margin and optimize treatment protocol selection.

**Materials and Methods:**

Ten‐phase‐gated four‐dimensional computed tomography (4DCT) scans of 14 patients with HCC were analyzed. For each patient, eight representative regions of interest (ROI) were delineated on each liver segment in all 10 phases. The coordinates of the center of gravity of each ROI were obtained for each phase, and then the respiratory motion in the left–right (LR), anteroposterior (AP), and craniocaudal (CC) directions was analyzed. Two sets of motion in each direction were also compared in terms of only two extreme phases and all 10 phases.

**Results:**

Motion of less than 5 mm was detected in 12 (86%) and 10 patients (71%) in the LR and AP directions, respectively, while none in the CC direction. Motion was largest in the CC direction with a maximal value of 19.5 mm, with significant differences between liver segment 7 (S7) and other segments: S1 (*p* < 0.036), S2 (*p* < 0.041), S3 (*p* < 0.016), S4 (*p* < 0.041), and S5 (*p* < 0.027). Of the 112 segments, hysteresis >1 mm was observed in 4 (4%), 2 (2%), and 15 (13%) in the LR, AP, and CC directions, respectively, with a maximal value of 5.0 mm in the CC direction.

**Conclusion:**

A significant amount of respiratory motion was detected in the CC direction, especially in S7, and S8. Despite the small effect of hysteresis, it can be observed specifically in the right lobe. Therefore, caution is required when using 4DCT to determine IM using only end‐inspiration and end‐expiration. Understanding the respiratory motion in individual liver segments can be helpful when selecting an appropriate treatment protocol.

## 1 INTRODUCTION

Liver cancer, predominantly in the form of hepatocellular carcinoma (HCC), is the sixth most common type of malignancy and the fourth leading cause of cancer‐related deaths worldwide.^1^ In recent years, stereotactic body radiation therapy (SBRT) and proton therapy (PT) for HCC have been reported effective.^2^, ^3^ To improve dose conformity on the target, the full use of image‐guided technology is necessary in addition to high‐precision irradiation technology, and measures to compensate for respiratory motion should be actively considered. Given the high recurrence rate of HCC, radiation exposure to a normal liver should be reduced as much as possible; thus, measures to compensate for respiratory motion should be established. However, despite the available strategies to reduce the effects of respiratory motion (e.g., respiratory‐gated irradiation, tracking irradiation, and breath‐hold irradiation)^4^, ^5^, ^6^; the respiratory motion of the target must still be determined. Four‐dimensional computed tomography (4DCT) has been recently used to measure target motion. The internal margin (IM) can be evaluated by using 4DCT in two extreme phases: end‐inspiration and end‐expiration, when respiratory motion is relatively small.^7^, ^8^, ^9^, ^10^ Kitamura et al. evaluated the details of the target motion in real time and detected hysteresis.^11^ In general, evaluation using the two phases of end‐inspiration and end‐expiration is based on the premise that the motion can be regarded as a linear motion. However, Kitamura et al. suggested that the motion is not actually a simple linear motion, but a so‐called hysteresis curve in which the trajectory is curved. Therefore, whether measurement occurring only during end‐inspiration and end‐expiration alone is sufficient to obtain the necessary target motion is yet to be fully determined. This problem may be due to the large volume of the liver and to significant individual differences.

Selection of the correct prescribed dose and treatment protocol is complicated by the liver's large volume and partial contact with the gastrointestinal tract. Currently in Japan, PT for HCC with a diameter of 4 cm or greater is covered by insurance, but other treatments are determined by the Japanese Society for Radiation Oncology unified protocol according to tumor location: peripheral, hilar, and gastrointestinal proximity.^12^ The choice of protocol is based on various diagnostic imaging criteria. However, the IM may be expanded due to the motion measurements obtained at the time of treatment planning CT imaging, and the distance between the target and the hepatic hilum or gastrointestinal tract may be shorter than initially expected. Thus, changing from a peripheral protocol to a gastrointestinal proximity protocol is common. Fraction size differs significantly between these protocols, and protocol significantly affects the treatment schedule and social activities of the patient. Thus, determining the amount of movement expected in each segment of the liver is crucial in selecting the most appropriate protocol between PT and photon therapy; this is generally a common problem in external radiation therapy. Tsai et al. attempted to determine the amount of respiratory motion for each liver segment,^10^ but only end‐inspiration and end‐expiration were evaluated, and did not report on the influence of hysteresis. Therefore, in this study, we investigated the amount of respiratory motion by each liver segment during all phases of respiration using 4DCT.

## 2 MATERIALS AND METHODS

### 2.1 Patient characteristics and imaging acquisition

In this study, 14 HCC (7 males, 7 females; median age, 62 years; range, 22−91 years) who received PT were enrolled. None of them had a history of surgery or irradiation nor clinical evidence of cirrhosis. This study was approved by the Institutional Review Board of our institution.

Patients were placed in the supine position and immobilized with a vacuum cushion with their arms over their heads. Respiratory pattern was neither mentioned nor coached so that the patients breathed freely with no alteration. 4DCT was performed to measure the amount of respiratory motion at the time of treatment planning CT imaging; no artifacts that could interfere with measurement were present. A laser displacement sensor, AZ‐733 V (Anzai Medical, Tokyo, Japan), was used to detect the displacement of a certain point near the patient's navel to obtain the respiratory waveform. The scan range included the entire liver during all respiratory phases. After scanning, a 10‐phase‐gated 4DCT image from 0% to 90% distributed over the whole respiratory cycle was constructed from the row 4DCT data. All CT images were imported into a treatment planning system, XiO‐M R 4.34.02 (Hitachi, Kashiwa, Japan), and registration software, Focal (Elekta, Stockholm, Sweden). Aquilion LB (Canon Medical Systems, Otawara, Japan) was used for the CT scans; images were taken in 2‐mm slices.

### 2.2 Measurement procedure

To calculate dose distribution, respiratory‐gated imaging was performed in which the imaging timing was adjusted during end‐exhalation. Next, to ensure accurate target contouring, three‐phase contrast‐enhanced CT was conducted, followed by 4DCT to measure respiratory motion. We adopted a method similar to that of Tsai et al. to evaluate respiratory motion for each liver segment, in which a virtual region of interest (ROI) is identified at the centroid of each liver segment.^10^

**FIGURE 1:**
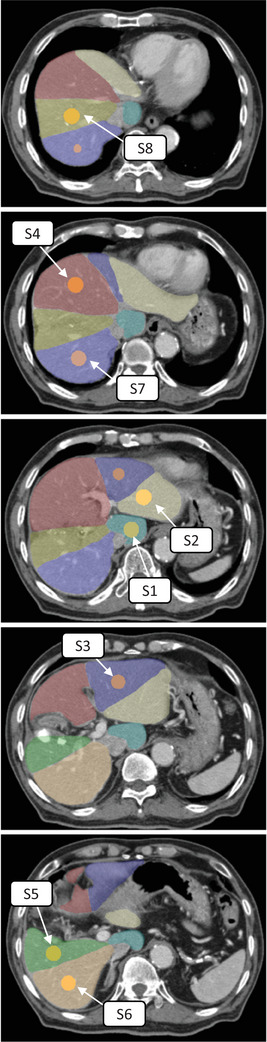
Example of each liver segment contouring and region of interest setting. Liver segment 1 (S1), S2, S3, S4, S5, S6, S7, and S8 showing according to the Couinaud classification.

**FIGURE 2:**
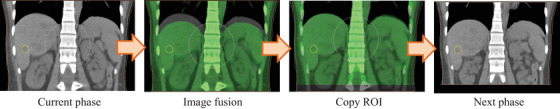
Copy procedure from one respiratory phase with the region of interest to the next respiratory phase.

Contouring for liver segments from liver segment 1 (S1) to S8 was performed according to the Couinaud classification by a single physician on a contrast‐enhanced CT image conducted for planning purposes (reference CT; refCT). A spherical ROI (eight in total) with a diameter of 15 mm was set in the center of each liver segment (Figure 1). The refCT fusion was repeated for the 4DCT images reconstructed for each 10% phase; then, the ROI on the refCT was copied in all 10 phases (Figure 2). Since local deformation of the liver due to respiration was occasionally observed, fusion was performed sequentially for each liver segment. The coordinates of the center of gravity of each ROI were obtained for each phase and the respiratory motion in the left–right (LR), anteroposterior (AP), and craniocaudal (CC) directions were analyzed. The correlation between the amount of motion of each liver segment and the right diaphragm in the CC direction was also assessed. Generally, the IM is determined based on motion during end‐inspiration and end‐expiration^10^; however, assuming that the target motion has a hysteresis curve trajectory, the motion information obtained from only two extreme phases may be insufficient. Therefore, two sets of motion in each direction were compared and evaluated taking into account both the extreme phases and all 10 phases. The amount of clinically relevant hysteresis was defined as the maximal distance between the different trajectories that the ROI follows during inhalation and exhalation. Wilcoxon's signed‐rank test was used for the statistical significance test. A *p*‐value of 0.05 was determined to be significant.

## 3 RESULTS

**FIGURE 3:**
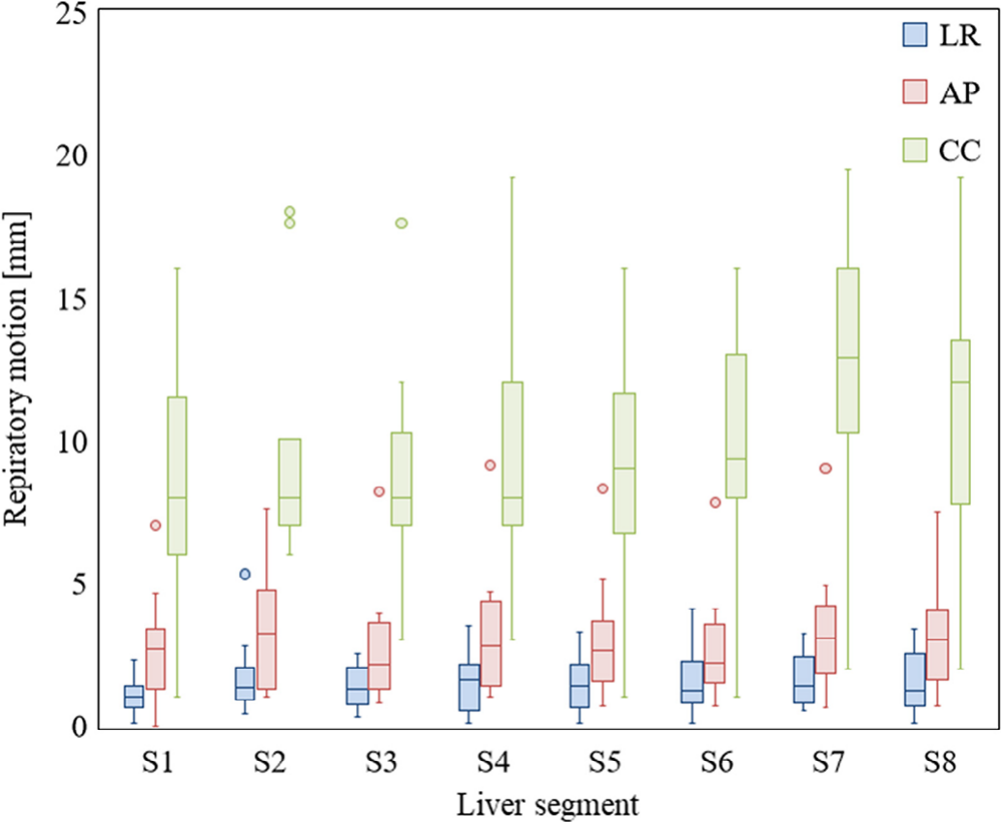
Box plot showing the directional respiratory motion of different liver segments. The median, minimum, and maximum values are presented with the first and third quartiles. LR = left–right, AP = anteroposterior, CC = craniocaudal.

**FIGURE 4:**
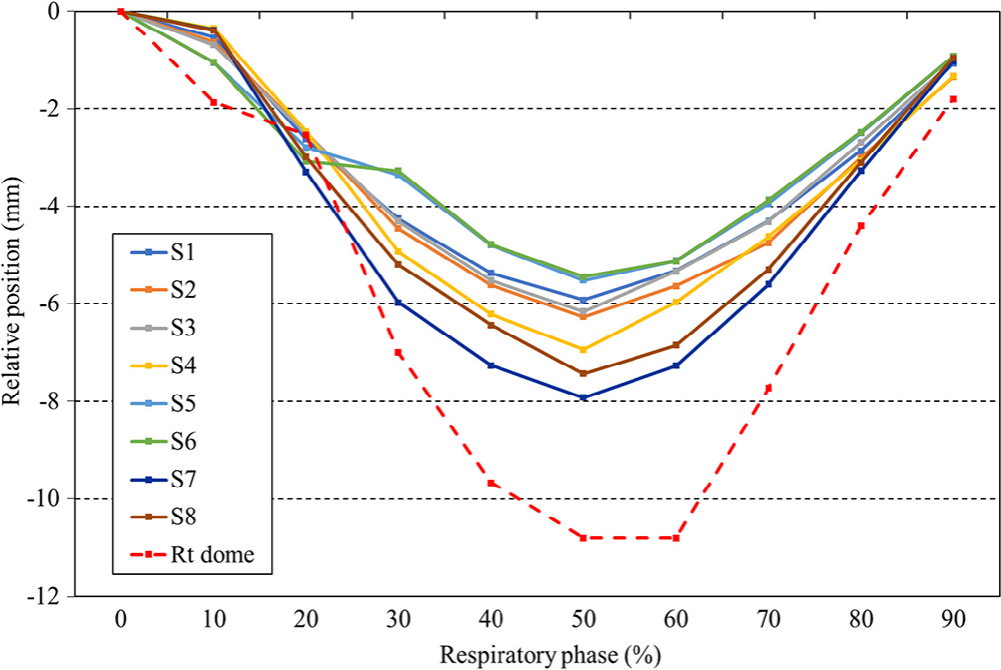
Phase‐by‐phase mean respiratory motion of each liver segment, and right diaphragm in the craniocaudal direction.

**FIGURE 5:**
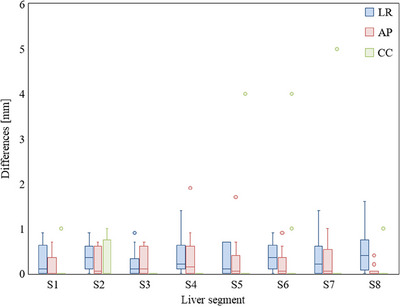
Box plot showing differences between the amount of motion during end‐inspiration and end‐expiration and the amount of motion in all 10 phases of different liver segments. The median, minimum, and maximum values are presented with the first and third quartiles. LR = left–right, AP = anteroposterior, CC = craniocaudal.

The average motion (mean ± standard deviation) was 1.5 ± 1.0 mm in the LR direction, 3.0 ± 2.0 mm in the AP direction, and 9.7 ± 4.2 mm in the CC direction. Figure 3 shows the results of the directional motions of each liver segment. Motion of less than 5 mm was detected in the LR and AP directions in 12 (86%) and 10 (71%) patients, respectively, and none in the CC direction. The motion was largest in the CC direction, with a maximal value of 19.5 mm. The LR and AP directions did not have significant differences between each segment. In the CC direction, motion in the S7 was significantly different from that in S1 (*p* < 0.036), S2 (*p* < 0.041), S3 (*p* < 0.016), S4 (*p* < 0.041), and S5 (*p* < 0.027). Figure 4 shows the correlation between the mean value of the motion of each liver segment in the CC direction and the right diaphragm for each phase. However, despite the differences in the motion of S4, S7, and S8, the proximity of each segment to the right diaphragm also affected the relative proximity of the amount of motion to that of the right diaphragm. Hysteresis >1 mm was detected in 10 patients (71%), but hysteresis >2 mm was detected in only two patients (14%). Figure 5 shows the difference between the amount of motion during end‐inspiration and end‐expiration, and the amount of motion when considering all 10 phases. Of the 112 segments analyzed in this study, hysteresis >1 mm was observed in 4 (4%), 2 (2%), and 15 (13%) in the LR, AP, and CC directions, respectively, with a maximal value of 5.0 mm in the CC direction.

## 4 DISCUSSION

Although SBRT and PT have been widely used for HCC treatment, respiratory motion must be considered to ensure effectiveness. However, accurate prediction of the amount of respiratory motion in the liver is difficult due to the lacking soft tissue contrast of image‐guided radiotherapy modalities, such as CT and cone‐beam CT. Fiducial markers and accumulated lipiodol are good indicators,^13^ but they are not always present at the appropriate positions. In addition, only two respiratory phases, end‐inspiration and end‐expiration, are typically used for the evaluation of respiratory motion, but the trajectory between them has not been sufficiently clarified. If the trajectory depicts a pronounced hysteresis curve, the amount of respiratory motion may be underestimated using only information on end‐inspiration and end‐expiration. In actual clinical practice, selection of different protocols depends on the localization of the tumor and can be changed due to the expanded IM with motion measurements obtained at the time of treatment planning CT imaging. Therefore, it is clinically significant to determine the respiratory motion in advance via both photon therapy and PT. Population‐based segmental trends may be helpful to accurately predict respiratory motion. In this study, we evaluated the relative tendencies of motion for each liver segment and compared the trajectory generated by analyzing two respiratory phases and the trajectories from all 10 phases.

Significant differences in the motion in the CC direction between S7 and other segments such as S1, S2, S3, S4, and S5 suggest that respiratory motion may differ between the right upper lobe and other lobes of the liver. The correlation between the motion of each liver segment in the CC direction and the right diaphragm motion weakened as the distance from the right diaphragm increased. The motion in the LR and AP directions tended to be less than 5 mm in many cases, which was smaller than in the CC direction. By contrast, some patients exhibited the same amount of motion in the AP direction as in the CC direction. Therefore, the AP direction has the largest variation among the three directions, suggesting that there may be individual differences, making it difficult to accurately estimate the population from limited analysis data. In S7 and S8, which are located near the right diaphragm, the amount of respiratory motion was particularly large. Our findings are consistent with those of Kitamura et al. in that the left lobe moves less than the right because the left lobe may be more rigidly fixed by the falciform ligament than the right lobe.^11^

In the present study, hysteresis was occasionally observed in the right lobe, where the amount of respiratory motion was large. However, hysteresis >2 mm was observed only in 2 of 14 cases (14%) and by segment only 3 of 112 sites (3%) in the CC direction. Kitamura et al. reported hysteresis >1 mm in 4 (20%) out of 20 patients, and a maximum of 4.0 mm was observed in the AP direction.^11^ Our results tend to have slightly larger hysteresis than their results. This may be due to our use a surrogate for the abdominal wall during 4DCT imaging, whereas Kitamura et al. used a fiducial marker implanted near the tumor as an index. In other words, the phase shift between the abdominal wall and each segment may be intervening as a modifier. Generally, 4DCT accurately acts as a surrogate for the movement of the abdominal wall, but the method itself may be an error factor, indicating that the extreme target positions must be determined by visual inspection directly instead of via breathing wave form, to avoid the incorrect selection of phases to generate composite internal target volume. Based on our results, respiratory motion estimation based on two extreme phases at end‐inspiration and end‐expiration is acceptable in most cases. However, especially in the right lobe, an error of several millimeters may occur in the CC direction due to hysteresis. Xi et al. reported that evaluation with only two extreme phases is sufficiently practical for a respiratory motion of 16 mm or less.^9^ However, our results did not support this finding because all patients in our study with hysteresis of at least 4 mm had a respiratory motion of approximately 13 mm. Conversely, those with motion >16 mm were observed in 8 of 112 segments, but without significant hysteresis. These data demonstrate large individual differences in respiratory motion, breathing pattern, and hysteresis. Therefore, respiratory motion values should be considered with caution. If IM is determined based on the results of only end‐inspiration and end‐expiration measurements, the 4DCT scans of all planes must be reviewed to avoid a geographic miss of the target. Treatment should also be carefully performed, and any contradiction with the results of 4DCT should be determined by confirming the motion of the right diaphragm with fluoroscopy before beginning treatment. If it is clinically permissible, in consideration of the motion uncertainties in the CC direction, an additional IM of several millimeters could be effective.

In addition to the large amount of respiratory motion in the liver, the nearby gastrointestinal tract may also affect the treatment policy as a result of the treatment plan simulation. Understanding the IM required for each liver segment is an important reference when determining indications and protocols. Therefore, the results of this study, which evaluated motion in the liver, will be helpful for determining final IM.

This study has some limitations. First, the amount of respiratory motion in each liver segment might have depended on pathological conditions (e.g., the progress of cirrhosis). Kitamura et al. reported that respiratory motion is large in cases with liver cirrhosis, and the tendency may be different in metastatic liver tumors in which the liver itself is in a normal state.^11^ However, as there was no evidence of cirrhosis in any patients we evaluated, we can rule out the effect of cirrhosis in this study. Moreover, the results of this study may not apply to patients with a history of surgery or irradiation or those who use an abdominal compression plate.^14^ Liver conditions in patients vary and may affect the amount of respiratory motion. Second, we addressed only the intrafractional motion to determine the intrafractional motion component of the internal target volume. In actual clinical practice, the influence of interfractional motion is significant, and countermeasures are being investigated.^15^ Future studies should investigate whether interfractional motion varies by liver segment. Finally, this study only analyzed the amount of motion of the center of gravity of the ROI set in each liver segment. During PT, the dose degradation due to motion is associated with the geometric motion from a single mass center and with changes in the water‐equivalent thickness (WET) along the entire beam path. The use of motion‐induced variation of WET or statistical methods to evaluate all voxel changes may hold higher significance in deciding or selecting internal margins.^16^, ^17^ These indicators cannot be uniquely determined because they depend on the beam arrangement, but considering such indicators for each liver segment is beneficial in PT, and this will be a concern in the future.

## 5 CONCLUSION

We used 4DCT to measure the amount of respiratory motion in individual liver segments in 14 patients with HCC. We detected a significant amount of respiratory motion in the CC direction, especially in S7 and S8. Respiratory motion tends to differ for each liver segment, with extensive variation between patients; therefore, individualized margins are desirable. In addition, the effect of hysteresis is small, caution is required especially in the right lobe when using 4DCT to determine IM using only end‐inspiration and end‐expiration. Understanding the respiratory motion in individual liver segments can contribute to the selection of an appropriate treatment protocol.

## AUTHOR CONTRIBUTIONS

**Takahiro Kato**: Conceptualization; investigation; methodology; writing—original draft. **Kimihiro Takemasa**: Resources. **Tomohiro Ikeda**: Methodology. **Hisanori Sakagami**: Formal analysis. **Ryohei Kato**: Methodology. **Yuki Narita**: Formal analysis. **Sho Oyama**: Validation. **Shinya Komori**: Resources. **Hisashi Yamaguchi**: Writing—review and editing. **Masao Murakami**: Writing—review and editing.

## CONFLICT OF INTEREST STATEMENT

The authors have no conflicts of interest to declare.

## ETHICAL APPROVAL

All procedures performed in studies involving human participants were in accordance with the ethical standards of the Institutional Review Board and with the 1964 Helsinki declaration and its later amendments or comparable ethical standards.
